# Audio-Visual Stress Classification Using Cascaded RNN-LSTM Networks

**DOI:** 10.3390/bioengineering9100510

**Published:** 2022-09-27

**Authors:** Megha V. Gupta, Shubhangi Vaikole, Ankit D. Oza, Amisha Patel, Diana Petronela Burduhos-Nergis, Dumitru Doru Burduhos-Nergis

**Affiliations:** 1 Department of Computer Engineering, New Horizon Institute of Technology and Management, University of Mumbai, Mumbai 400615, Maharashtra, India; 2 Department of Computer Engineering, Datta Meghe College of Engineering, University of Mumbai, Mumbai 400708, Maharashtra, India; 3 Department of Computer Sciences and Engineering, Institute of Advanced Research, Gandhinagar 382426, Gujarat, India; 4 Department of Mathematics, Institute of Technology, Ahmedabad 382481, Gujarat, India; 5 Faculty of Materials Science and Engineering, Gheorghe Asachi Technical University of Iasi, 700050 Iasi, Romania

**Keywords:** stress, emotion, action units, speech, audio visual, RNN-LSTM

## Abstract

The purpose of this research is to emphasize the importance of mental health and contribute to the overall well-being of humankind by detecting stress. Stress is a state of strain, whether it be mental or physical. It can result from anything that frustrates, incenses, or unnerves you in an event or thinking. Your body’s response to a demand or challenge is stress. Stress affects people on a daily basis. Stress can be regarded as a hidden pandemic. Long-term (chronic) stress results in ongoing activation of the stress response, which wears down the body over time. Symptoms manifest as behavioral, emotional, and physical effects. The most common method involves administering brief self-report questionnaires such as the Perceived Stress Scale. However, self-report questionnaires frequently lack item specificity and validity, and interview-based measures can be time- and money-consuming. In this research, a novel method used to detect human mental stress by processing audio-visual data is proposed. In this paper, the focus is on understanding the use of audio-visual stress identification. Using the cascaded RNN-LSTM strategy, we achieved 91% accuracy on the RAVDESS dataset, classifying eight emotions and eventually stressed and unstressed states.

## 1. Introduction

Today, 82 percent of Indians are stressed, as per the Cigna 360 Well-being study [1]. Rising stress levels even led to India being named the world’s most depressed country a few years ago. In our society, the word “stress” has become overused. There are many more people suffering from stress in India than in any Western nation, despite the fact that the issue has long been dismissed as a “western” problem. More and more Indians are experiencing stress, depression, anxiety, and other related conditions due to factors such as work pressure, life’s challenges, relationships, financial stress, or mental overload. The fact that stress is one of the main factors in cardiac arrests, blood pressure increases, and an increased risk of chronic diseases at an early age is even more shocking. A recent study by the World Health Organization and the Global Burden of Disease Study found that since the COVID-19 pandemic hit, there has been an increase in stress and anxiety among people [2]. Our problems have only gotten worse as a result of the global pandemic. Experts are concerned about a recent development that suggests we may be on the verge of a terrible mental illness pandemic—not to mention the emotional and financial toll the COVID-19 crisis has taken on many. There is still a lack of mental health awareness and a continuing stigmatization of mental illnesses in our country. Despite the internet and open conversations initiated by celebrities, there are many myths, taboos, and pieces of misinformation surrounding mental health. It is important to remember that going to therapy, seeing a psychiatrist, or being open about your feelings does not make you weak or bad. Mental illnesses should not be stigmatized, and getting the right help at the right time can prevent a lot of problems.

### 1.1. Causes of Mental Stress

Stress is a condition of mental pressure for individuals facing problems relating to environmental and social well-being which leads to many diseases. It was discovered that academic exams, human relationships, interpersonal difficulties, life transitions, and career choices all contribute to stress. Such stress is commonly associated with psychological, physical, and behavioral issues [3].

According to Lazarus and Folkman (1984), “stress is a mental or physical phenomenon formed through one’s cognitive appraisal of the stimulation and is a result of one’s interaction with the environment”. The existence of stress depends on the existence of the stressor. Feng (1992) and Volpe (2000) defined a stressor as “anything that challenges an individual’s adaptability or stimulates an individual’s body or mentality”. Stress can be caused by environmental factors, psychological factors, biological factors, and social factors, as shown in Table 1.

### 1.2. Importance of Mental Stress Detection

Human stress represents an imbalanced state [4] of an individual and is triggered when environmental demands exceed the regulatory capacity of the individual [5]. Because of its unhealthy effects [6], stress detection is an ongoing research topic among both psychologists and engineers, and has been applied to lie detection tests [7], emergency call identification [8], and the development of better human–computer interfaces [9]. People experience stress because of the requests and pressures put on them. The situation becomes more difficult when they perceive the circumstances to be overpowering and believe that it will be difficult to adapt [10]. Three levels of stress can be distinguished depending on the time of exposure to stressors. Acute stress is the innate ‘‘flight-or-fight” response in the face of short-lasting exposure to stressors, and it is not considered harmful. Episodic stress appears when stressful situations occur more frequently, but they cease from time to time. It is associated with a very stressful and chaotic life [11]. Finally, chronic stress, which is the most harmful, takes place when stressors are persistent and long-standing, such as family problems, job strain, or poverty [12]. To prevent stress reaching the highest level and help diminish the risks [13], it is necessary to detect and treat it in its earlier stages, i.e., when it is still acute or episodic stress. Stress identification has gained remarkably high attention in various fields in the last two decades. These fields include the medical sector, forensics, smart environments, teaching, learning and education, human–computer interactions, the emergency services, and of course real-time situations, which are the most crucial [14]. The identification of stress is a standout among the best research topic points for psychologists as well as engineers. Stress management should begin before stress starts to cause medical problems. This is where stress monitoring can help. In recent years, interest in artificial intelligence-aided health monitoring or psychological counseling systems has increased due to the convenience and efficiency of machine learning-based algorithms. To provide appropriate services in these areas, the mental state of the user must be detectable. Among various emotional states, we focus on a methodology to detect the user’s stress status.

### 1.3. Role of Emotion in Stress Identification

Emotions are present in almost every decision and moment of our lives. Thus, recognizing emotions awakens interest, since knowing what others feel helps us to interact with them more effectively. Emotions are considered a psychological state [15]. In the process of detecting stress using the audio-visual approach, it is important to detect the emotional state of the person. Hence, emotion recognition should be performed to decide on the stress level. The field of emotion recognition (ER) is a part of human–computer interaction, and this field has evolved very rapidly in the last decade. Several works have been performed on emotion recognition using audio and video; however, recent work has been completed on the fusion of the different modalities. Expressing emotions while interacting with others has always been a major part of communication among humans. Emotions are reflected through voice, facial expressions, and hand gestures, and can easily transcend the boundary of languages. A lot of work has been performed over the decades on automatic emotion recognition as part of human–computer interaction.

**Figure 1:**
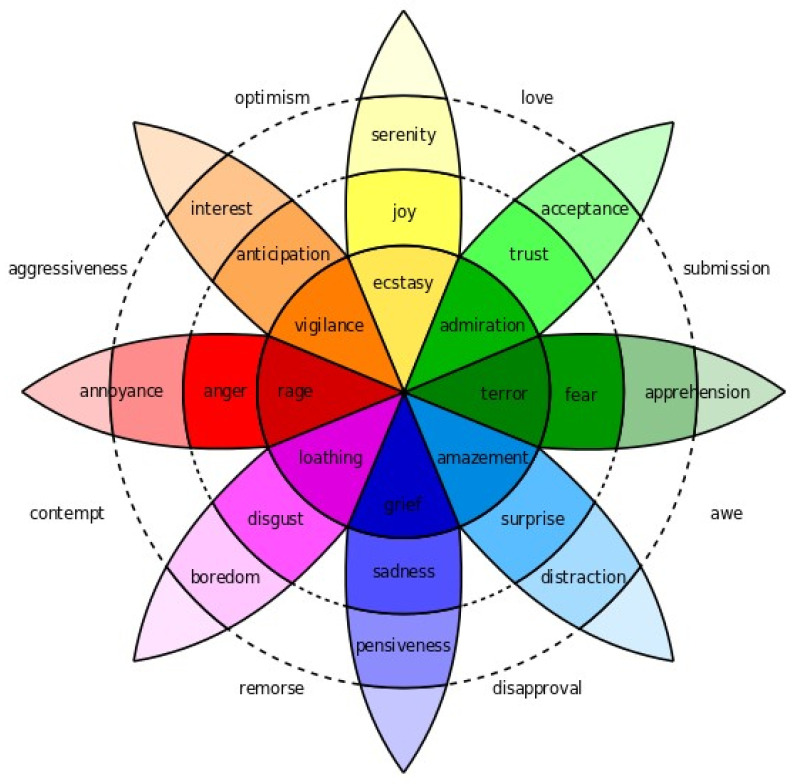
Plutchik wheel of emotion.

It must be considered that emotions are subjective to an individual, i.e., each subject may experience a different emotion in response to the same stimuli. Thus, emotions can be classified into two different models—the discrete model and the dimensional model. The discrete model includes basic emotions such as happiness, sadness, fear, disgust, anger, surprise, and mixed emotions such as motivation (thirst, hanger, pain, mood), self-awareness (shame, disgrace, guilt), etc. The dimensional model is expressed in terms of two emotions, valence (disgust, pleasure) and arousal (calm, excitement). The various emotions experienced by a human can be represented through the Plutchik wheel of emotion [16], as shown in Figure 1.

**Figure 2:**
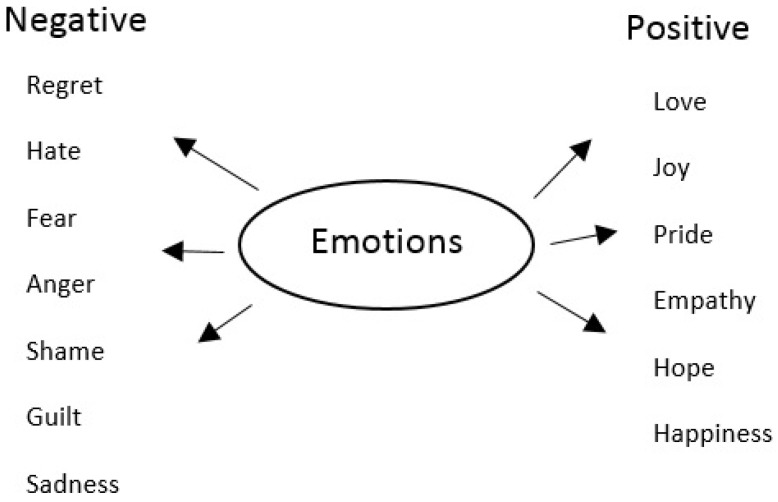
Positive and negative emotions.

Several researchers have analyzed human stress using basic emotions. It is possible to map emotions with the stress level. Stress can be detected based on emotions obtained from the audio-visual data. Human emotions are expressed in the voice as well as on the face. The emotional state is extracted from the audio-visual data first. Positive emotions such as happiness, joy, love, pride, and pleasure can have a positive effect, such as improving daily work performance, and negative emotions such as anger, terrible, sad and, disgust can have a negative impact on the health of a person. Positive and negative emotions are represented in Figure 2. Emotional signs such as depression, terrible, unhappiness, anxiety, agitation, and anger are responsible for stress. Stress can be detected from the two emotional states of anger and disgust.

**Figure 3:**
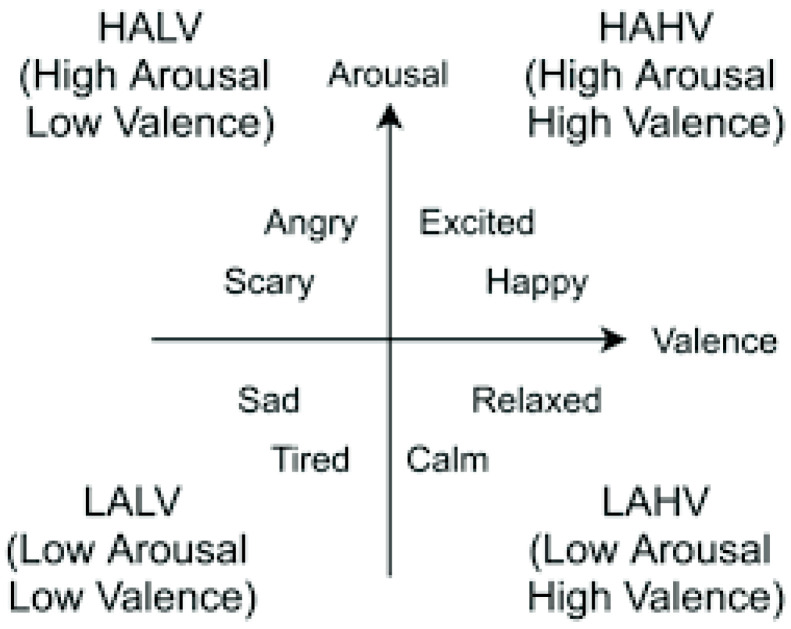
Arousal–valence model for emotion representation.

The valence–arousal space, as illustrated in Figure 3, can be subdivided into four quadrants, namely low arousal/low valence (LALV), low arousal/high valence (LAHV), high arousal/low valence (HALV), and high arousal/high valence (HAHV) [17].

Our model reached an accuracy of 91% on the ‘The Ryerson Audio-Visual Database of Emotional Speech and Song (RAVDESS), outperforming some of the previous solutions evaluated in similar conditions. As far as we know, our study also represents the first attempt to combine speech and facial expressions to recognize the eight emotions in RAVDESS and finally conclude on a stressed or relaxed state.

The rest of the paper is organized as follows: Section 2 describes preceding research studies related to our proposal. Section 3 presents feature extraction, elaborating on the facial action coding system (FACS) and OpenFace. Section 4 summarizes the dataset and the proposed methodology. Section 5 describes the main results obtained and performance analysis. Finally, in Section 6, we discuss the main conclusions of our study and propose future research lines.

## 2. Literature Review

By outlining some of the difficulties that these systems encountered, we present earlier automatic stress detection techniques here. We describe the stress-inducing stimuli that were employed, how stress was measured, the signals that were gathered, and the machine learning techniques that were applied in these studies.

### 2.1. Stress Detection Using Speech Signal

Stress detection from speech signals has many applications. It is used in psychology to monitor the different stress levels of patients with different stress conditions and provide necessary treatments. The safety and security of a system can be established by monitoring the different stress levels of pilots, deep sea divers and military officials undertaking law enforcement. Stress detection is also useful in speaker identification, deception detection and identification of threatening calls in a few cases of crimes [18]. In order to effectively express his or her message, a person must choose the words to use at each stage of speaking. These choices, as well as the language, syntax, and timing of speech, can all be impacted by stress [19,20]. These changes in wording, grammar, and timing can then be employed as vocal cues to indicate stress. Other changes are also brought on by stress, though. For instance, in order to create sound waves during speaking, the body modifies the tension of many muscles to push air through the vocal folds and out of the vocal tract [21]. Stress raises the breathing rate and muscle tension, which alters speech mechanics and, as a result, the way speech sounds [22,23]. A voice-based stress detection system, named StressSense [24], was implemented on Android phones to detect the stress levels from human voice. The stress model was developed in several contexts, testing various speakers and events.

Kevin Tomba et al. [25] worked on the Berlin Emotional Database (EMO-DB), the Keio University Japanese Emotional Speech Database (KeioESD) and the Ryerson Audio-Visual Database of Emotional Speech and Song (RAVDESS). SVM and ANN algorithms were used. It was found that mean energy, mean intensity, and Mel frequency cepstral coefficients proved to be good features for speech analysis. However, only audio signals were considered, and not audio-visual data. N.P. Dhole and S.N. Kale investigated RNN classification and used it on the BERLIN and HUMAINE Datasets. They also used Audacity software to build real datasets for recurrent neural network applications. Despite being effective for audio signals, the efficiency percentage was not calculated [14]. Audio-visual data were not considered. Mansouri et al. [26] used a wavelet and neural network to create and implement an emotion identification system from speech signals. EMO-DB and SAVEE were used. The accuracy was judged to be satisfactory. The procedure, however, was time-consuming. The detection of stress was not taken into account.

### 2.2. Stress Detection Using Audio-Visual Data

Speech and facial expression are two natural and effective ways of expressing emotions when human beings communicate with each other. During the last two decades, audio-visual emotion recognition integrating speech and facial expression has attracted extensive attention owing to its promising potential applications in human–computer interaction [27,28]. However, recognizing human emotions with computers is still a challenging task because it is difficult to extract the best audio and visual features characterizing human emotions.

G. Giannakakis et al. [29] recorded videos using a camera. The videos’ facial cues were used to identify signs of anxiety and stress. This method achieved good classification accuracy. However, a 1 min video duration could yield more reliable estimates. Kah Phooi et al. [30] used the eNTERFACE and RML (RAVDESS) datasets. They used a combined rule-based and machine learning approach for emotion recognition using audio-visual data. Anupam Agrawal and Nayaneesh Kumar Mishra [31] used SAVEE and created their own dataset. They worked on emotion classification based on the fusion of audio and visual data. However, it was found that the results can be improved using deep learning techniques. Noroozi, F et al. [32] worked on audio-visual emotion recognition in video clips. The datasets used were SAVEE, eNTERFACE’05, and RML.

Audio-visual data were not considered for the stress detection, and only audio signals were used. Moreover, the accuracy of the results can be improved. Although there is much research discussing the recognition and analysis of the six basic emotions, i.e., anger, disgust, fear, happiness, sadness, and surprise, considerably less research has focused on stress and anxiety detection from audio visuals, as these states are considered as complex emotions that are linked to basic emotions (e.g., fear). The results of emotion state recognition from audio-visual data can be improved using deep learning techniques, which can be further used to detect stress.

### 2.3. Analysis

Overall, this seems to be an interesting area of research, and the analysis of the existing work would help in carrying out future research. Table 2 provides an overview of numerous studies reflecting the same area of interest, together with the datasets they employed, the techniques used, the pros of these techniques, and the scope for advancement.

To sum up, despite the fact that other works in the literature also performed multimodal emotion recognition on RAVDESS, such as Wang et al. [33], who used facial images to generate spectrograms, which were then used for data augmentation to improve the SER model performance in six emotions, our work is the first that, to our knowledge, detects the stressed and relaxed state using the audio-visual information of RAVDESS by means of aural and facial emotion recognition using the eight emotions.

### 2.4. Transition from Holistic Facial Recognition to Deep Learning Based Recognition

In the 1990s and 2000s, the face recognition community was dominated by holistic techniques. Faces are represented using holistic approaches utilising the complete facial region. Many of these approaches function by projecting facial photographs into a low-dimensional space that eliminates unimportant features and variances. PCA is one of the most prominent techniques in this field. Deep neural networks trained with extremely huge datasets have lately supplanted older approaches based on hand-crafted features and typical machine learning techniques. Deep face recognition algorithms, which employ hierarchical design to learn discriminative face representation, have significantly enhanced state-of-the-art performance and spawned a multitude of successful real-world applications. Deep learning employs many processing layers to discover data representations with numerous feature extraction levels [34,35].

## 3. Feature Extraction from Facial Expressions

### 3.1. Facial Action Coding System

Eckman and Friesen [36] created the FACS technique to analyze facial microexpressions and identify the emotions of the persons being studied. It is predicated on the notion that various facial muscle patterns can be linked to various emotions, and that the face areas where these muscles are active can be used to identify an individual’s emotion. The fundamental benefit of FACS over other face analysis techniques is that it can detect concealed emotions, even when the person is attempting to imitate other emotions. People’s emotions are frequently assessed using FACS.

**Figure 4:**
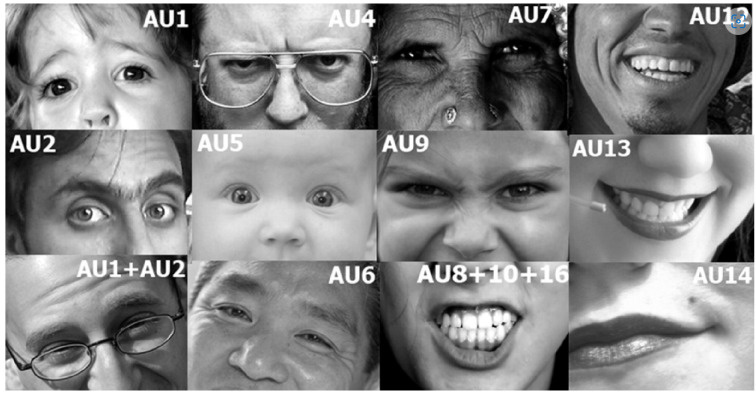
Action units.

FACS divides the face into 46 action units (AUs), as shown in Figure 4, which can be either nonadditive (an AU’s activity is unrelated to the activity of other AUs) or additive (when one AU is activated, it causes another AU or group of AUs to activate). The Action units along with their pictorial representation is depicted in Table 3 below. The Facial Action Coding System (FACS) is used to classify human facial movements according to how they appear on the face. FACS encodes the movements of specific facial muscles from slight instantaneous changes in facial appearance.

Almost any anatomically conceivable facial expression can be coded using FACS, which breaks it down into the specific action units (AUs) that give rise to the expression, as shown in Table 4. FACS is a widely used method for accurately describing facial expressions. Facial expressions are considered as the signal, and emotions as the message.

### 3.2. OpenFace

OpenFace is a tool intended for computer vision and machine learning researchers, the affective computing community, and people interested in building interactive applications based on facial behavior analysis. OpenFace is the first toolkit capable of facial landmark detection, head pose estimation, facial action unit recognition, and eye-gaze estimation with available source code for both running and training the models. Specifically, OpenFace can identify AUs 1, 2, 4, 5, 6, 7, 9, 10, 12, 14, 15, 17, 20, 23, 25, 26, 28 and 45.

There are two ways to categorize AUs: intensity and presence. Presence (for instance, AU01 c) indicates whether an AU is visible on the face. On a scale of 1 to 5, intensity indicates the degree of AU intensity (min to max). Both of these scores are presented by OpenFace.

These two scores are provided by OpenFace. The output file’s column AU01 c encodes 0 as not present and 1 as present for the presence of AU 1. The output file’s column AU01 r has continuous values in the range of 0 (not present), 1 (present at minimum intensity), and 5 (present at maximum intensity) for the intensity of AU 1.

## 4. Proposed Method to Classify Mental Stress

Our proposed stress detection framework includes two systems: a speech emotion recognizer and a face emotion recognizer. The outputs of these subsystems were integrated to identify the dominant emotion and eventually result in a stressed or unstressed state. In the current research, we made a point to highlight a novel method of implementing two different algorithms to function better than any single algorithm working individually. The proposed algorithm not only improves the overall accuracy in determining emotions but also is faster than each individual algorithm, as it uses the advantages of each algorithm and eliminates the disadvantages or time-consuming processes of each of them. Further, the work may seem complicated at the first glance; however, the accuracy improvement in the field of mental stress determination is what we are looking for, and our set objectives for the research work are met through the approach.

### 4.1. The RAVDESS Dataset

The Ryerson Audio-Visual Database of Emotional Speech and Song (RAVDESS) is licensed under CC BY-NA-SC 4.0. The paper by Livingstone SR and Russo FA (2018) described the construction and validation of the dataset.

There are 7356 files in the RAVDESS. Each file was rated ten times for emotional validity, intensity, and authenticity. A group of 247 people who were typical untrained adult research participants from North America provided ratings. The second group of 72 people provided test–retest data. Emotional validity, interrater reliability, and test–retest intra-rater reliability were all reported to be high.

#### 4.1.1. Description

The dataset included all 7356 RAVDESS files in their entirety (total size: 24.8 GB). The three modality formats for each of the 24 actors were audio-only (16 bit, 48 kHz.wav), audio-video (720p H.264, AAC 48 kHz,.mp4), and video-only (480p H.264, AAC 48 kHz,.mp4) (no sound). Please take note that Actor 18 did not have any song files.

#### 4.1.2. Data

A total of 4948 samples were used for this task. Audio files were extracted from video-audio files using the “mp4 to wav” algorithm. The filenames for each of the 7356 RAVDESS files were distinctive. A seven-part numerical identifier comprised the filename (e.g., 02-01-06-01-02-01-12.mp4). These codes specified the properties of the stimulus:

The filename identifiers used are illustrated in Table 5 below.

Taking the example of the RAVDESS filename 02-01-06-01-02-01-12.mp4:

Video-only (02)

Speech (01)

Fearful (06)

Normal intensity (01)

Statement “dogs” (02)

1st Repetition (01)

12th Actor (12)

Female, as the actor ID number is even.

### 4.2. Proposed System

#### 4.2.1. Why RNN?

ANN and/or CNN have been presented before in the literature, and an accuracy of around 80% has been reported for them. In our literature review, we did not find any individual algorithm which would improve the accuracy of prediction beyond 90%. So, we needed a different approach wherein we combined two relatively less processor-heavy algorithms to work on and improve the accuracy and simultaneously work at a faster rate. However, as rightly pointed out by the reviewer, in our continued plan for our research work, we will make a point to work on ANN- and CNN-based algorithms to either present a comparative analysis or to cascade them as per our intended method to verify their performance for the said cause. Recurrent neural networks (RNNs) have been successfully applied to sequence learning issues such as action identification, scene labeling, and language processing. An RNN has a recurrent connection, unlike feed-forward networks such as convolutional neural networks (CNNs), where the previous hidden state is an input to the subsequent state. An enhanced RNN, or sequential network, called a long short-term memory network, allows information to endure. It is capable of resolving the RNN’s vanishing gradient issue. Persistent memory is achieved via a recurrent neural network or RNN. Let us imagine that when reading a book or viewing a movie, you are aware of what happened in the preceding scene or chapter. RNNs function similarly; they retain the knowledge from the past and apply it to process the data at hand. Due to their inability to remember long-term dependencies, RNNs have this drawback. Long-term dependency issues are specifically avoided when designing LSTMs.

In our case, RNN is used to classify data of facial landmark position with respect to time for visual data analysis and to classify the pitch of different frequencies of the audio signal with respect to time to determine the emotions.

Speech and facial expressions are used to detect users’ emotional states. These modalities are combined by employing two independent models connected by a novel approach. By merging the information from aural and visual modalities, audio-visual emotion identification is vital for the human–machine interaction system. We propose a cascaded RNN-LSTM approach for audio-visual emotion recognition through correlation analysis. The emotions will finally be categorized as a stressed mental state or a relaxed mental state. We use the RAVDESS dataset for the verification of the proposed algorithm.

#### 4.2.2. Speech-Based Stress Detection

**Figure 5:**
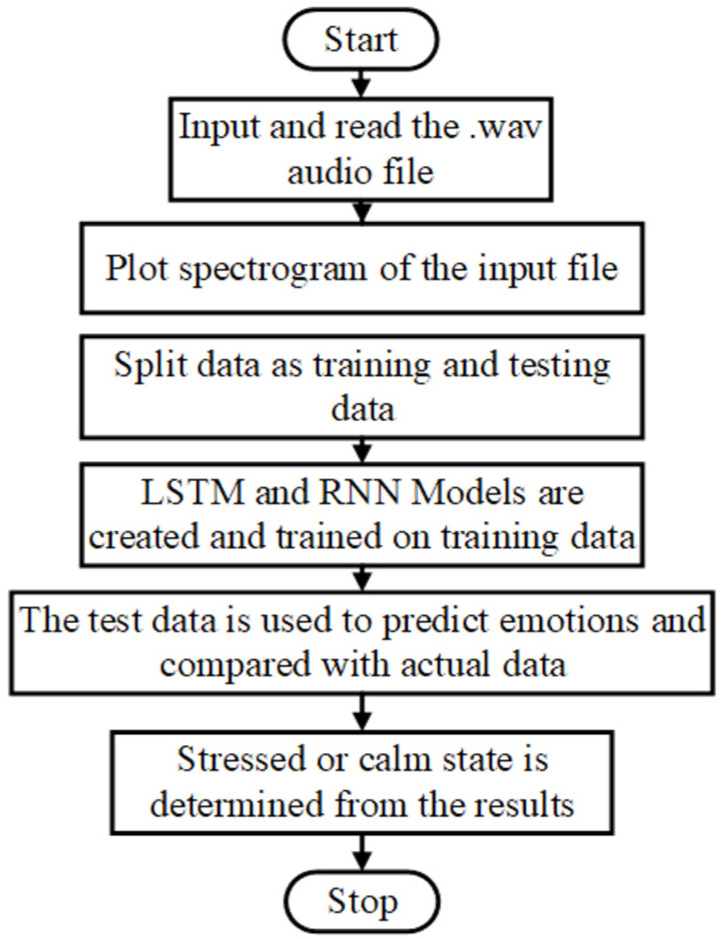
Flowgraph for stress recognition using speech signals.

**Figure 6:**
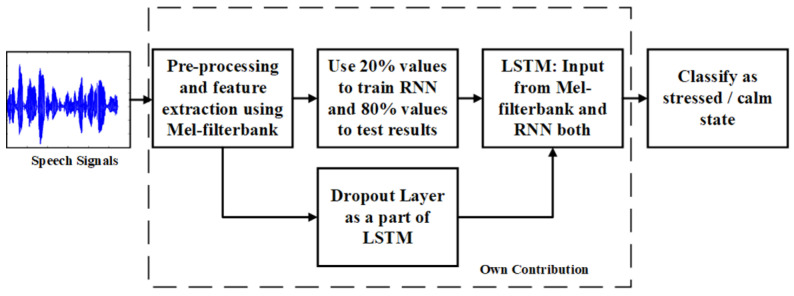
Speech Signals based Human Stress detection.

The flowgraph for stress recognition using speech signals is shown in Figure 5. In the proposed approach, two closely related ML algorithms viz. RNN and LSTM are cascaded together, as shown in the flowchart (Figure 6). Cascading improves the convergence time of the combined algorithm. The Mel-frequency cepstral coefficient (MFCC) is the most well-known spectral feature, since it is used to model the human auditory perception system. Here, speech signals are pre-processed and filtered using MFCC. The features of the input signal are extracted at this stage. These features are sent to 4 neurons RNN and 10 neuron LSTM working in parallel with each other. The RNN module which does not have a cell state generates the required labels for these features while the LSTM module is used for emotion prediction only. The LSTM module receives labels from the RNN module as its first input and the extracted features from MFCC as its second input. This combined approach reduces the size of the LSTM module by reducing the number of neurons required for emotion prediction, e.g., a 40-neuron LSTM module is replaced by a 10-neuron LSTM module with a 4-neuron RNN module to achieve the same result at a faster rate. Moreover, to prevent the model from overfitting, the LSTM module employs a dropout layer by randomly setting other edges of the hidden layer to zero. This reduces the convergence time to about 3/4 of the traditional approach.

#### 4.2.3. Proposed Method for Audio-Visual Based Stress Detection

**Figure 7:**
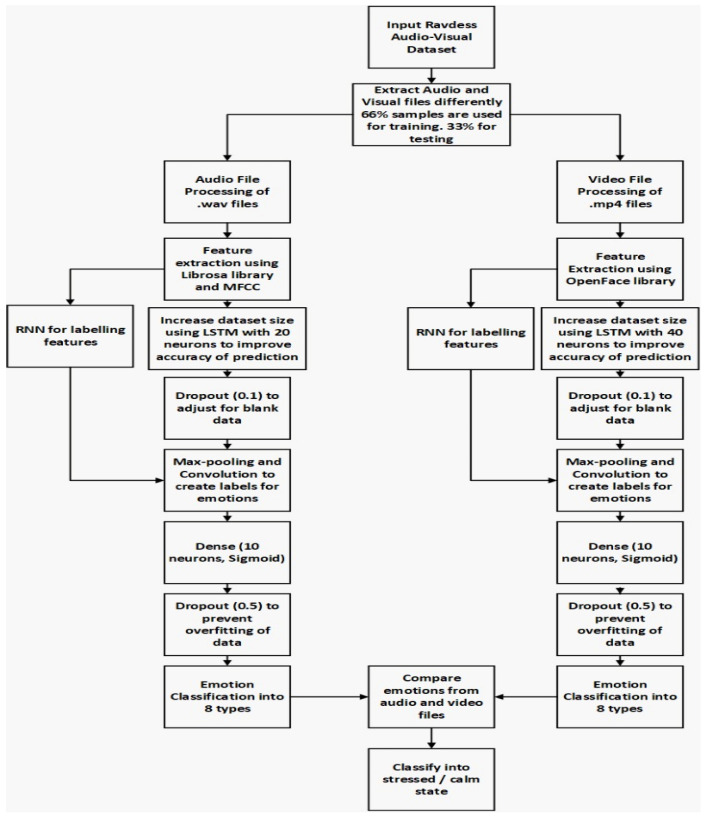
Proposed system workflow.

Our deep learning model contains two individual input streams, i.e., the audio network processing audio signals with the cascaded RNN-LSTM model, and the visual network processing visual data with the hybrid RNN-LSTM model. The flowchart for the algorithm is in Figure 7 below.

In the proposed algorithm, audio files are extracted from the video files and processed separately. Librosa is used to process audio files while OpenFace is used to process video files. Overall, 66% of samples are used for training purposes, while the rest are used for testing the algorithm. In the algorithm, RNN and LSTM work parallelly to improve the speed of the feature extraction process. Audio signals need 20 neurons in the LSTM network while video signals need 40 neurons due to their signal processing requirements. MFCC is used as a filter for feature extraction. Dropout layers are used to prevent data from overfitting. Max pooling with convolution creates the final 8 required labels from the features. A dense sigmoid function is used for the final classification of the output with 10 neurons each. The separate outputs of both audio and video files are compared on a common platform to improve the accuracy by matching the missing labels. The following emotions are predicted in this model: “neutral”: “01”, “calm”: “02”, “happy”: “03”, “sad”: “04”, “angry”: “05”, “fearful”: “06”, “disgust”: “07”, “surprised”: “08”. Finally, 8 emotions are classified into 2 mental states—stressed and relaxed. First of all, we chose the method of comparing both audio and video files to avoid any misrepresentation of emotions due to the use of only one kind of file. In a scenario where the classification of both files is different, the average sum of scores of each signal will determine the probability of the inclination of the signals to a particular emotion. However, such a scenario has not yet occurred in our work, and hence the algorithm has not yet been validated.

### 4.3. Analysis

We used the Jupyter interface to run the program. LibROSA, a python package, was used for music and audio analysis, while the OpenFace package was used for facial motion tracking.

**Figure 8:**
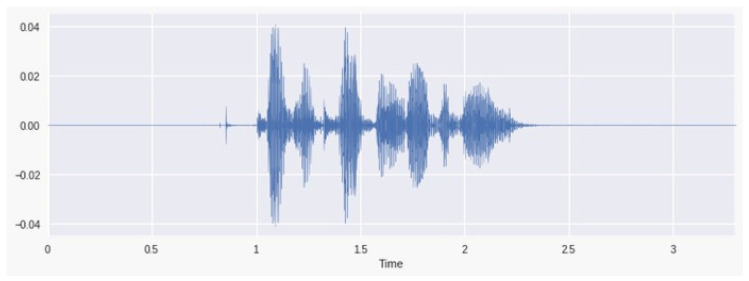
Audio signal.

We plotted the signal from a random file with audio and facial recognition separated as shown in Figure 8 below.

**Figure 9:**
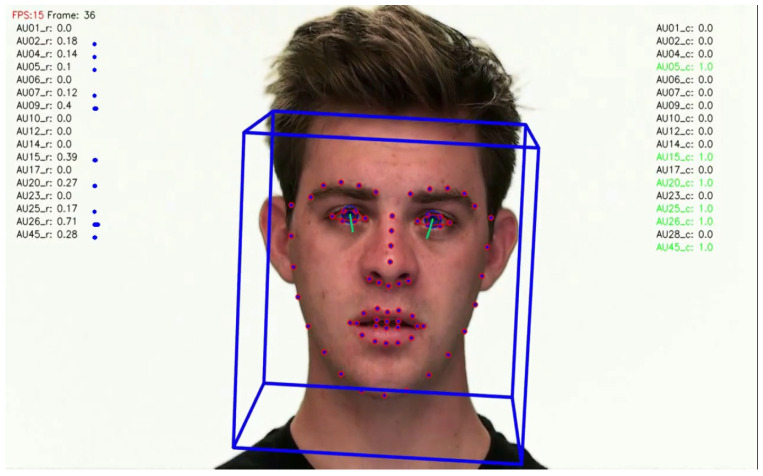
Facial recognition example 1.

**Figure 10:**
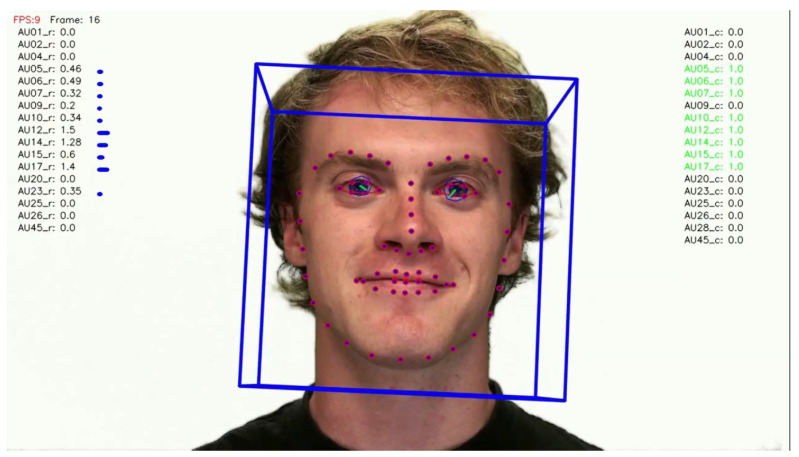
Facial recognition example 2.

Two facial recognition examples are illustrated in Figure 9 and Figure 10 for frames 36 and 16 respectively.

## 5. Experimental Results

NumPy array was created for extracting Mel-frequency cepstral coefficients (MFCCs), while the classes for prediction were extracted from the name of the file.

To apply the cascaded RNN-LSTM method effectively, we need to expand the dimensions of our array, adding a third one using the NumPy “expand_dims” feature.

Layer (type) Output Shape Param #

=================================================================

conv1d_1 (Conv1D) (None, 40, 128) 768

_________________________________________________________________

activation_1 (Activation) (None, 40, 128) 0

_________________________________________________________________

dropout_1 (Dropout) (None, 40, 128) 0.1

_________________________________________________________________

max_pooling1d_1 (MaxPooling1 (None, 5, 128) 0

_________________________________________________________________

conv1d_2 (Conv1D) (None, 5, 128) 82,048

_________________________________________________________________

activation_2 (Activation) (None, 5, 128) 0

_______________________________________________________________

dropout_2 (Dropout) (None, 5, 128) 0.5

_________________________________________________________________

flatten_1 (Flatten) (None, 640) 0

_________________________________________________________________

dense_1 (Dense) (None, 10) 6410

_________________________________________________________________

activation_3 (Activation) (None, 10) 0

=================================================================

Total params: 89,226

Trainable params: 89,226

Non-trainable params: 0

**Figure 11:**
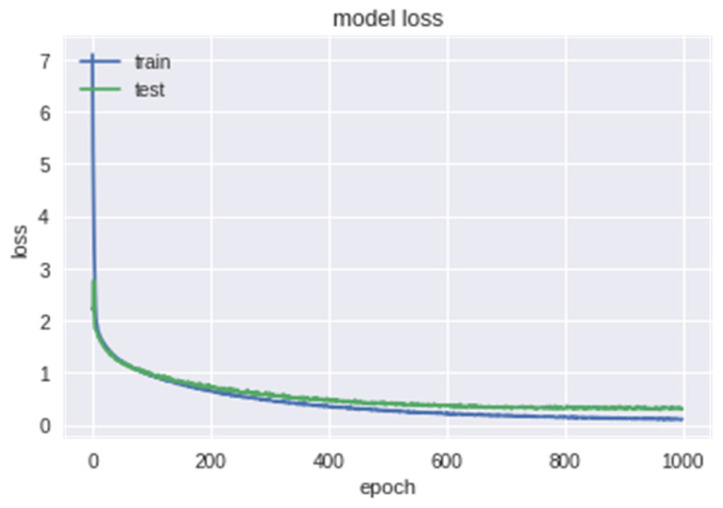
Loss of epochs based on training and test data.

**Figure 12:**
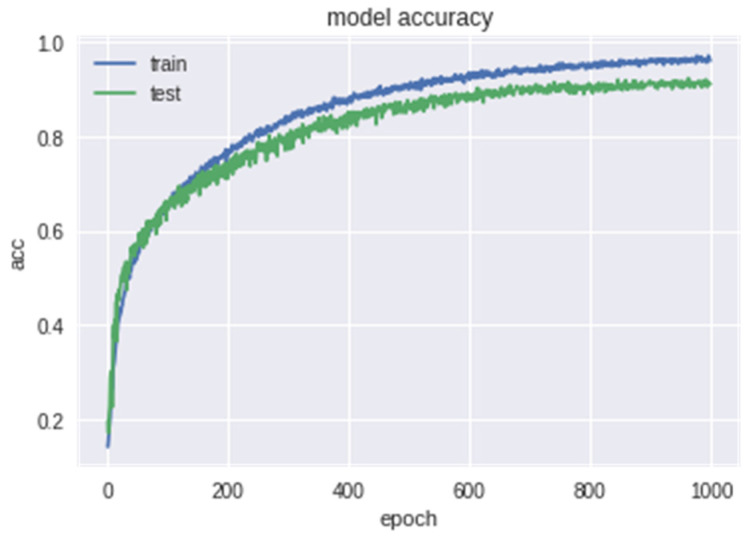
Accuracy of the Model.

The model loss of epochs based on training and test data is shown in the Figure 11 below. Figure 12 indicates the accuracy of the model.

**Figure 13:**
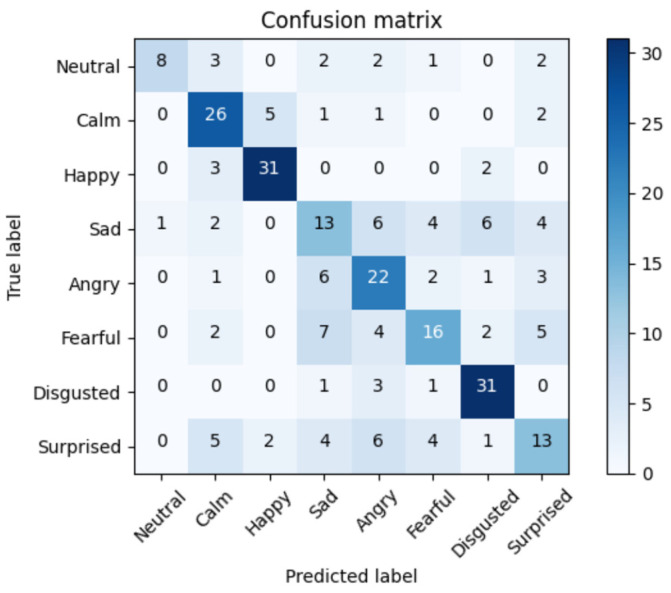
Average confusion matrix for the SVM algorithm. Accuracy = 76%.

**Figure 14:**
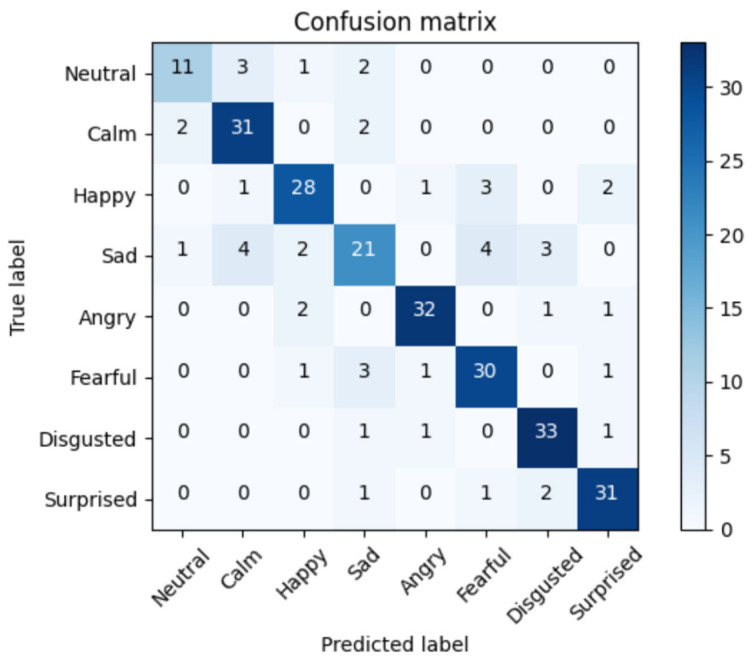
Average confusion matrix for the LSTM Algorithm. Accuracy = 82%.

**Figure 15:**
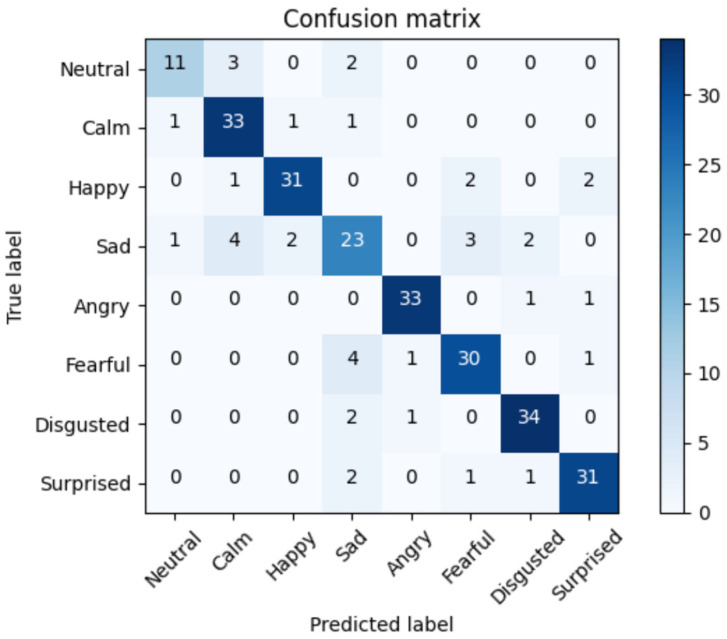
Average confusion matrix for RNN-LSTM approach. Accuracy = 91%.

To understand the errors of the top solution, we extracted the confusion matrix of the SVM, LSTM, and RNN-LSTM approaches with an accuracy of 76%, 82%, and 91%, respectively. The confusion matrix displayed in the Figure 13, Figure 14 and Figure 15 below is the rounded average value of the errors and the correct predictions obtained from the folds of the 5-CV. This matrix will display an average of 288 samples (1440/5).

Figure 15 reveals that the RNN-LSTM approach showed a good performance, except for some samples. The ‘Sad’ class contained the highest number of errors, mistaking this class in most cases for other emotions such as ‘Disgusted’ or ‘Fearful’, although it also confused this emotion with ‘Calm’, which may be caused by the low arousal level of both emotions.

The proposed algorithm is compared with the conventional ones and the performance analysis is presented the Table 6.

Final Output:

1633/1633 [==============================]—0s 125s/step

Accuracy: 91.00%

The existing work was focused on either audio or facial images. In audio-visual data, the separate output of audio and video files was compared on a common platform to improve accuracy by matching the missing labels. In order to enhance the accuracy further, we increased the dimensions of the dataset, as LSTM works better with more data. The accuracy for prediction for the proposed algorithm for the RAVDESS dataset is 91%.

## 6. Our Contributions

Only image-based classification may give polarized results in cases where the image under processing lacks the overall gesture being conveyed. Moreover, using audio and visual signals will help to improve the emotion classification accuracy, which is needed to determine whether the algorithm further needs to be fully developed for the medical determination of mental stress. Although we used well-established packages for our work, we made several changes to the algorithm to make it work and provide novelty. The changes in the algorithm include cascading or the parallel operation of algorithms (which usually runs sequentially), the addition of dropout layers to adjust the blank values and to avoid overfitting of the data, and processing of both audio and video files to compare and improve classification accuracy. We would like to state that this method of implementing the algorithm has never been reported in the literature before.

## 7. Conclusions and Future Scope

Detecting stress is essential before it turns chronic and leads to health issues. The current paper suggests that audio-visual data have the potential to detect stress. In our society, stress is becoming a major concern, and modern employment challenges such as heavy workloads and the need to adjust to ongoing change only make the situation worse. In addition to severe financial losses in businesses, people are experiencing health issues related to excessive amounts of stress. Therefore, it is crucial to regularly check your stress levels to detect stress in its preliminary stages and prevent harmful long-term consequences. The necessity for individuals to handle chronic stress gave rise to the concept of stress detection. The accuracy of the cascaded RNN-LSTM approach for the RAVDESS dataset is 91%. The obtained results are 15–20% better than those of other conventional algorithms. The proposed method is an excellent starting point to work towards mental health by detecting stress and improving one’s quality of life.

The evaluation of the test results showed that the successful detection of stress is achieved, although further improvements and extensions can be made. The implementation of this system can be improved by using more efficient data structures and software to reduce delays and achieve real-time requirements.

## Figures and Tables

**Table 1:** Major causes of stress.

**Cause of Stress**	Interpersonal conflict
	Role conflict
	Career concern
	Occupational demands
	Work overload
	Poor working condition
	Lack of social support
	Lack of participation in decision making

**Table 2:** Analysis of earlier work in the same field of study.

Title	Datasets Used	Technique	Pros of Technique	Scope for Improvement
Stress Detection Through Speech Analysis Kevin Tomba et al. (ICETE 2018) [25]	Berlin Emotional Database (EMO-DB), the Keio University Japanese Emotional Speech Database (KeioESD), and the Ryerson Audio-Visual Database of Emotional Speech and Song (RAVDESS)	SVMs and ANNs were chosen.	MFCCs, mean energy, and the mean intensity were all demonstrated to be effective speech analysis features.	Only audio input was considered and not audio-visual data
Study of Recurrent Neural Network Classification of Stress Types in Speech Identification N.P. Dhole, S.N. Kale (IJCSE 2018) [14]	BERLIN and HUMAINE Datasets	RNN	Real time dataset was created	Efficiency percentage not calculated. Works only on audio and not audio-visual data
Designing and Implementing of Intelligent Emotional Speech Recognition with Wavelet and Neural Network Mansouri et al. (IJACSA 2016) [26]	Datasets: EMO-DB and SAVEE	Artificial neural network	Accuracy is good	Time-consuming method. Stress detection was not considered
Stress and anxiety detection using facial cues from videos G. Giannakakis et al. (Elsevier 2017) [29]	Recorded using camera	Using facial cues from the videos	Achieves good classification accuracy	1 min video duration could yield more reliable estimates
A Combined Rule-Based & Machine Learning Audio-Visual Emotion Recognition Approach Kah Phooi et al. (IEEE 2016) [30]	eNTERFACE and RML(RAVDESS)	Emotion recognition using rule-based and machine learning	Fusion of audio and visual data	Worked only on emotion using audio-visual data, stress was not detected
Fusion-based Emotion Recognition System Anupam Agrawal, Nayaneesh Kumar Mishra (IEEE 2016) [31]	SAVEE and also created own dataset	SVM for emotion classification	Fusion of audio and visual data	Results can be improved using deep learning techniques
Audio-Visual Emotion Recognition in Video Clips Noroozi, F et al. (IEEE 2016) [32]	SAVEE eNTERFACE’05 and RML	Fusion at the decision level	Comparison of results based on all 3 datasets	Stress detection was not considered

**Table 3:** Action units.

1	Inner Brow Raiser	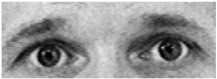
2	Outer Brow Raiser	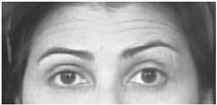
4	Brow Lowerer	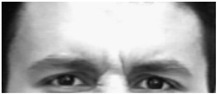
5	Upper Lid Raiser	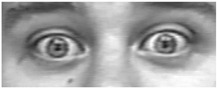
6	Cheek Raiser	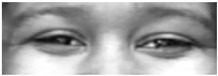
7	Lid Tightener	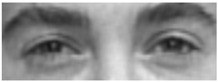
9	Nose Wrinkler	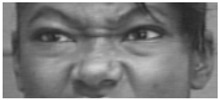
10	Upper Lip Raiser	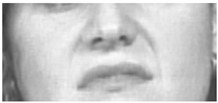
11	Nasolabial Deepener	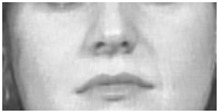
12	Lip Corner Puller	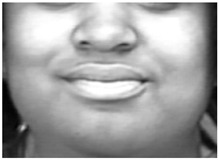
13	Cheek Puffer	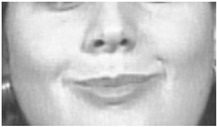
14	Dimpler	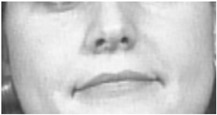
15	Lip Corner Depressor	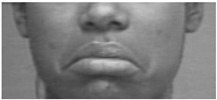
16	Lower Lip Depressor	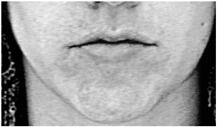
17	Chin Raiser	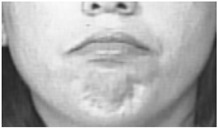
18	Lip Puckerer	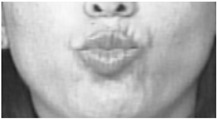
20	Lip stretcher	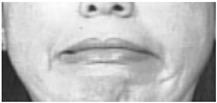
22	Lip Funneler	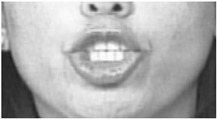
23	Lip Tightener	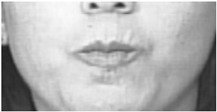
24	Lip Pressor	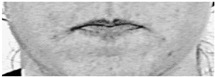
25	Lips part	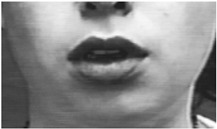
26	Jaw Drop	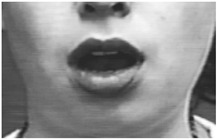
27	Mouth Stretch	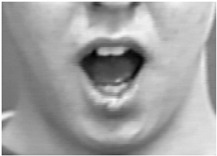
28	Lip Suck	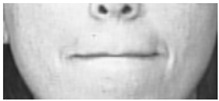
41	Lid droop	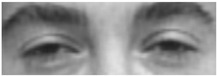
42	Slit	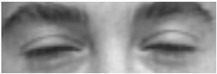
43	Eyes Closed	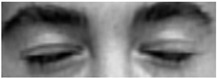
44	Squint	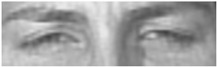
45	Blink	

**Table 4:** List of AUs involved in basic expressions.

Basic Expressions	Involved Action Units
Surprise	AU 1, 2, 5, 15, 16, 20, 26
Fear	AU 1, 2, 4, 5, 15, 20, 26
Disgust	AU 2, 4, 9, 15, 17
Anger	AU 2, 4, 7, 9, 10, 20, 26
Happiness	AU 1, 6, 12, 14
Sadness	AU 1, 4, 15, 23

**Table 5:** Identifiers of RAVDESS filenames.

Identifier	Coding Description of Factor Levels
Modality	01 = Audio-video, 02 = Video-only, 03 = Audio-only
Channel	01 = Speech, 02 = Song
Emotion	01 = Neutral, 02 = Calm, 03 = Happy, 04 = Sad, 05 = Angry, 06 = Fearful, 07 = Disgust, 08 = Surprised
Intensity	01 = Normal, 02 = Strong
Statement	01 = “Kids are talking by the door”, 02 = “Dogs are sitting by the door”
Repetition	01 = First repetition, 02 = Second repetition
Actor	01 = First actor, …… 24 = Twenty-fourth actor

**Table 6:** Performance analysis of the proposed system on RAVDESS.

Classification Accuracy %	SVM	RNN	MFCC (LSTM)	MFCC(LSTM+RNN) Proposed Algorithm
Neutral	100	70	90	100
Calm	66	85	86	98
Happy	86	83	84	93
Sad	81	75	78	86
Angry	89	84	91	98
Fearful	70	72	74	87
Disgust	73	70	75	82
Surprise	60	75	78	84
Overall Accuracy	76	78	82	91

## Data Availability

Not applicable.
